# Requirements for resuming translation in chimeric transfer-messenger RNAs of *Escherichia coli* and *Mycobacterium tuberculosis*

**DOI:** 10.1186/1471-2199-15-19

**Published:** 2014-09-15

**Authors:** Iwona K Wower, Christian Zwieb, Jacek Wower

**Affiliations:** 1 Department of Animal Sciences, Auburn University, Auburn, AL 36849, USA; 2 Department of Biochemistry, University of Texas Health Science Center at San Antonio, San Antonio, TX 78229, USA

**Keywords:** Chimeric tmRNA, SmpB, *Trans*-translation, Protein tagging

## Abstract

**Background:**

*Trans*-translation is catalyzed by ribonucleprotein complexes composed of SmpB protein and transfer-messenger RNA. They release stalled ribosomes from truncated mRNAs and tag defective proteins for proteolytic degradation. Comparative sequence analysis of bacterial tmRNAs provides considerable insights into their secondary structures in which a tRNA-like domain and an mRNA-like region are connected by a variable number of pseudoknots. Progress toward understanding the molecular mechanism of *trans*-translation is hampered by our limited knowledge about the structure of tmRNA:SmpB complexes.

**Results:**

Complexes consisting of *M. tuberculosis* tmRNA and *E. coli* SmpB tag truncated proteins poorly in *E. coli*. In contrast, the tagging activity of *E. coli* tmRNA is well supported by *M. tuberculosis* SmpB that is expressed in *E. coli*. To investigate this incompatibility, we constructed 12 chimeric tmRNA molecules composed of structural features derived from both *E. coli* and *M. tuberculosis*. Our studies demonstrate that replacing the hp5-pk2-pk3-pk4 segment of *E. coli* tmRNA with the equivalent segment of *M. tuberculosis* tmRNA has no significant effect on the tagging efficiency of chimeric tmRNAs in the presence of *E. coli* SmpB. Replacing either helices 2b-2d, the single-stranded part of the ORF, pk1, or residues 79–89 of *E. coli* tmRNA with the equivalent features of *M. tuberculosis* tmRNA yields chimeric tmRNAs that are tagged at 68 to 88 percent of what is observed with *E. coli* tmRNA. Exchanging segments composed of either pk1 and the single-stranded segment upstream of the ORF or helices 2b-2d and pk1 results in markedly impaired tagging activity.

**Conclusion:**

Our observations demonstrate the existence of functionally important but as yet uncharacterized structural constraints in the segment of tmRNA that connects its TLD to the ORF used for resuming translation. As *trans*-translation is important for the survival of *M. tuberculosis*, our work provides a new target for pharmacological intervention against multidrug-resistant tuberculosis.

## Background

**Figure 1:**
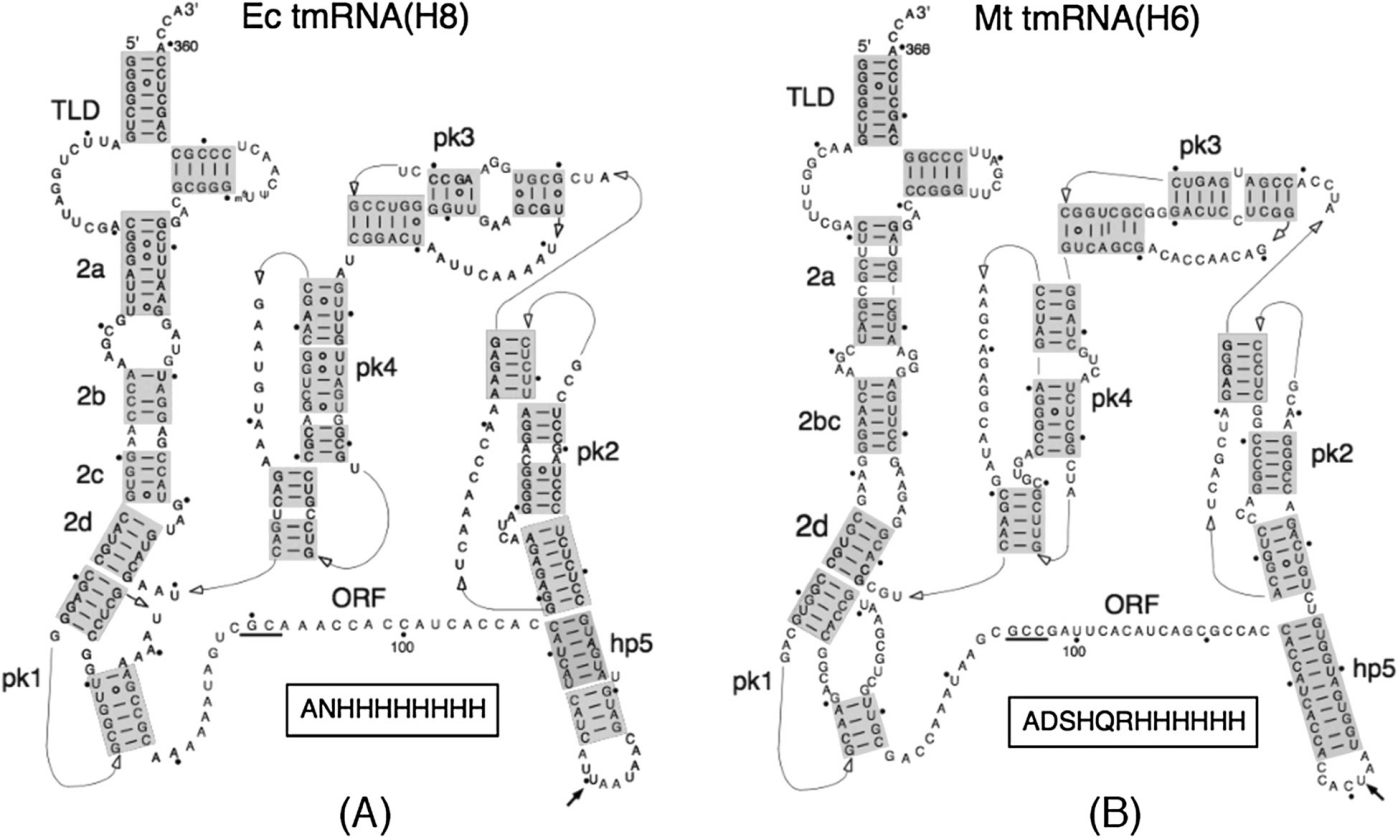
**Secondary structures of mutant tmRNAs from *E. coli *and *M. tuberculosis*. (A)** Ec tmRNA(H8). **(B)** Mt tmRNA(H6). The 5’-to-3’ direction of the tmRNA chain is indicated by lines with open arrowheads. Nucleotides are labeled with dots in increments of ten. Four pseudoknots, the open reading frame, the tRNA-like domain, and helix 5 are denoted pk1-pk4, ORF, TLD, and hp5, respectively. Four segments of helix 2 are shown as 2a-2d. Resume codons GCA and GCC are underlined. An arrow marks the stop codon. Mutant peptide tags ANHHHHHHHH and ADSHQRHHHHHH are highlighted.

Translation of mRNAs that are missing stop codons stalls ribosomes and produces truncated proteins. To recycle stalled ribosomes and degrade defective proteins, bacteria use *trans*-translation, a quality control process mediated by a ribonucleoprotein particle composed of transfer-messenger RNA (tmRNA) and SmpB [1,2]. The tmRNA acts as both tRNA and mRNA through its tRNA-like domain (TLD) and a mRNA-like region (MLR) with an open reading frame (ORF) that encodes a short proteolysis-inducing tag peptide [3-5]. In the majority of bacterial species, the TLD and MLR segments are connected by four pseudoknots (pk1-pk4) (Figure 1). TmRNA binding to stalled ribosomes is facilitated by elongation factor Tu (EF-Tu), SmpB and ribosomal protein S1. The SmpB molecule mimics the D and anticodon arms that are absent in tmRNA [6]. EF-Tu binds to the T-arm of the TLD precisely as observed in canonical aminoacyl-tRNAs [7,8]. Protein S1 was found in *E. coli* tmRNA:ribosome complexes that were assembled *in vivo*[9]. S1 has been shown to bind to free tmRNAs and tmRNA:SmpB complexes by contacting the MLR, pk2 and pk3 [10]. Although essential for *trans*-translation in *M. tuberculosis*, protein S1’s contributions to *trans*-translation in *E. coli* and other bacteria remain poorly understood [11-14].

Cryo-electron microscopy (cryo-EM) studies yielded a number of snapshots of the tmRNA:SmpB complex as it binds to the A site of the stalled ribosome and is accommodated in the P site [15-20]. The TLD interacts with the ribosome similarly to a canonical tRNA whereas pk2-pk4 form an arc around the head of the 30S subunit. While tmRNA maintains its overall structure during translocation from the A to the P site, the region encompassing helices 2a-2d (hp2a-2d) and pk1 undergoes significant conformational changes. Molecular modeling suggests that pk1 unfolds, at least partially, as the TLD is translocated to the E site [21]. This suggestion is consistent with our earlier studies demonstrating that pk1 could be reduced to a hairpin without affecting tmRNA’s tagging activity [22]. Although the resolution of cryo-EM images is not sufficiently high for observing subtle changes in the single stranded regions of the ribosome-bound tmRNA molecule, site-directed mutagenesis experiments revealed that the conserved nucleotides (85-UAG-86) located upstream of the ORF together with SmpB are instrumental for setting the correct reading frame on *E. coli* tmRNA [23]. According to more recent cryo-EM studies, SmpB contacts at least five nucleotides upstream of the resume codon [18].

In this article, we explore structure-function relationships in tmRNA:SmpB complexes using chimeric tmRNA molecules that have been constructed by swapping equivalent segments of *E. coli* and *M. tuberculosis* tmRNAs. Although these tmRNAs have very similar predicted secondary structures, *E. coli* SmpB very poorly supports the tagging activity of *M. tuberculosis* tmRNA on *E. coli* ribosomes. In contrast, *M. tuberculosis* SmpB promotes the tagging activity of *E. coli* tmRNA to nearly the same level as the *E. coli* SmpB. These findings provided an opportunity to systematically scan the tagging activity the tmRNA with the aim to identify incompatibilities with the tagging activity of the heterologous *M. tuberculosis* tmRNA: *E. coli* SmpB complex. Our studies demonstrate that replacing the hp5-pk2-pk3-pk4 segment of *E. coli* tmRNA with the equivalent segment of *M. tuberculosis* tmRNA produces a chimeric molecule that efficiently tags truncated proteins in the presence of *E. coli* SmpB. Swapping helices 2b-2d, pk1 and the single-stranded segment upstream of the ORF separately yields chimeric tmRNA:*E. coli* SmpB complexes with a decreased tagging activity. In contrast, replacing *E. coli* tmRNA segments composed either of helices 2b-2d and pk1 or of pk1 and single-stranded sequence upstream of the ORF with equivalent segments of *M. tuberculosis* tmRNA yields chimeric tmRNAs that tag very poorly in the presence of *E. coli* SmpB. Since SmpB does not interact with helices 2b-2d and pk1, our findings suggest the existence of an as yet uncharacterized tmRNA feature that plays an important role in *trans*-translation.

## Results

### *In vivo* tagging by *E. coli* and *M. tuberculosis* tmRNAs

SmpB protein binds to the TLD to form a complex that mimics the shape of canonical tRNA [6]. Cryo-EM studies of the *E. coli* TLD:SmpB complex accommodated in the P site suggest that conserved residues 134–140 (and possibly 18–24) of SmpB are in close proximity to five conserved tmRNA residues (positions 85–89) [18]. Given that the ORF and the pseudoknots of tmRNA can be extensively modified without affecting *trans*-translation [24,25], one would expect that tmRNA molecules of similar size and secondary structure, like the canonical tRNAs, are active in heterologous *trans*-translational systems. To test this conjecture, we used tmRNA(H8), a fully functional derivative of *E. coli* tmRNA in which the ORF has been modified to contain eight histidine codons (Figure 1A). *In vivo*, tmRNA(H8) tags truncated ribosomal protein L27 with a histidine-rich polypeptide (ANH_8_) to yield a fusion protein that is resistant to proteolysis and therefore can be easily detected in fractionated *E. coli* lysates by staining with Coomassie Blue or by Western blot analysis [24]. To investigate the tagging activities of the *M. tuberculosis* tmRNA, a similar construct, Mt tmRNA(H6), that encodes the protease resistant ADSHQRH_6_ tag was synthesized (Figure 1B). The plasmid-encoded Mt tmRNA(H6) was expressed in *E. coli* cells as efficiently as Ec tmRNA(H8) [24].

**Figure 2:**
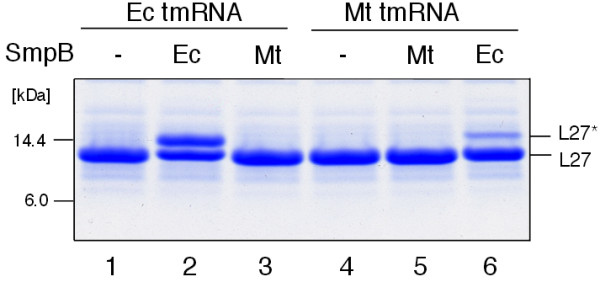
***In vivo *tagging of truncated ribosomal protein L27 by *E. coli *tmRNA(H8) and *M. tuberculosis *tmRNA(H6).***E. coli* IW764 cells were transformed with plasmids pWOW-Δ/E (lane 1), pWOW-E/E (lane 2), pWOW-M/E (lane 3), pWOW-Δ/M (lane 4), pWOW-M/M (lane 5) and pWOW-E/M (lane 6). Lysates of IPTG-induced *E. coli* IW764 cells harboring plasmid pWOW derivatives were fractionated on a 12.5% SDS-polyacrylamide gel. Tagged proteins were detected by staining the gel with Coomassie Blue. L27 and L27* denote truncated and tagged ribosomal protein L27, respectively. Positions of 6.0 and 14.4 kDa molecular markers are shown on the left side of the gel image.

The main components of our *in vivo* tagging system are six variants of plasmid pWOW (Additional file 1: Figure S1 and Table S1) that express tmRNA-directed His-tagged truncated L27 in the presence of protein SmpB. Consistent with our previous studies, the plasmid-encoded *E. coli* tmRNA(H8) and SmpB efficiently tagged protein L27 in IW764, an *E. coli* strain lacking both the *ssrA* gene (which encodes tmRNA) and the *smpB* gene (Figure 2, lane 2) [24]. In contrast, the tagging of truncated protein L27 by *M. tuberculosis* tmRNA(H6) and *E. coli* SmpB was largely ineffective (Figure 2, lane 6). No tagging of truncated L27 was observed in *E. coli* IW764 that was transformed with pWOW derivatives encoding *M. tuberculosis* SmpB (Figure 2, lanes 3 and 5).

**Figure 3:**
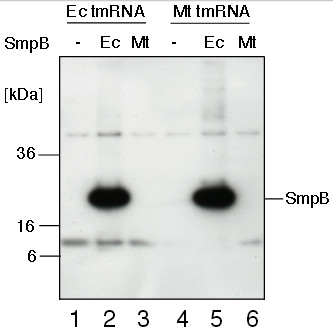
**Detection of *E. coli *and *M. tuberculosis *SmpB proteins in *E. coli *IW764 cells used in the *in vivo *tagging assay illustrated in Figure**2**.** Lanes 1-6 are as in Figure 2. Both SmpB proteins had C-terminal epitope Tag-100. Lysates of IPTG-induced *E. coli* IW764 cells harboring plasmid pWOW derivatives were fractionated on a 12.5% SDS-polyacrylamide gel. SmpB proteins were visualized by Western blotting with anti-Tag-100 antibodies. Positions of 6, 16 and 36 kDa molecular markers are shown on the left side of the gel image.

Further studies using Northern blotting revealed that the mRNAs for *E. coli* and *M. tuberculosis* SmpB were expressed well in *E. coli* IW764 cells (data not shown). However, while *E. coli* SmpB protein was efficiently translated, no *M. tuberculosis* SmpB protein could be detected in cell lysates using Western blotting (Figure 3, lanes 3 and 6). This finding was unexpected because *M. tuberculosis* SmpB cloned in a plasmid vector under the control of the T7 promoter can be efficiently overexpressed in *E.coli* IW764 strain (see Methods). Translation of *M. tuberculosis* SmpB was likely repressed by inhibitory RNA structures encompassing the ribosome-binding site in its mRNA. Such structures were predicted to form only in *M. tuberculosis* SmpB mRNAs that were transcribed under control of its natural promoter [26].

### *In vivo* tagging activity of chimeric tmRNAs

**Figure 4:**
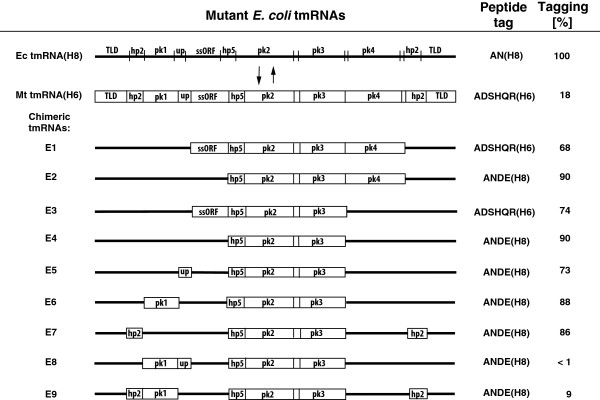
**Mutant *E. coli *tmRNAs. ***E. coli* tmRNA structure is represented by a bold line. Segments of *M. tuberculosis* tmRNA are shown as boxes in hybrid tmRNA species. Tagging efficiencies of hybrid tmRNA derivatives were compared to the tagging efficiency of *E. coli* tmRNA(H8). Four pseudoknots, the open reading frame, the tRNA-like domain, helix 2 and helix 5 are denoted pk1-pk4, ORF, TLD, hp2 and hp5, respectively. The A-rich single-stranded segment connecting pk1 and ORF is marked as ‘up’. All tagging activities are shown as the mean of triplicate determinants with standard error.

**Figure 5:**
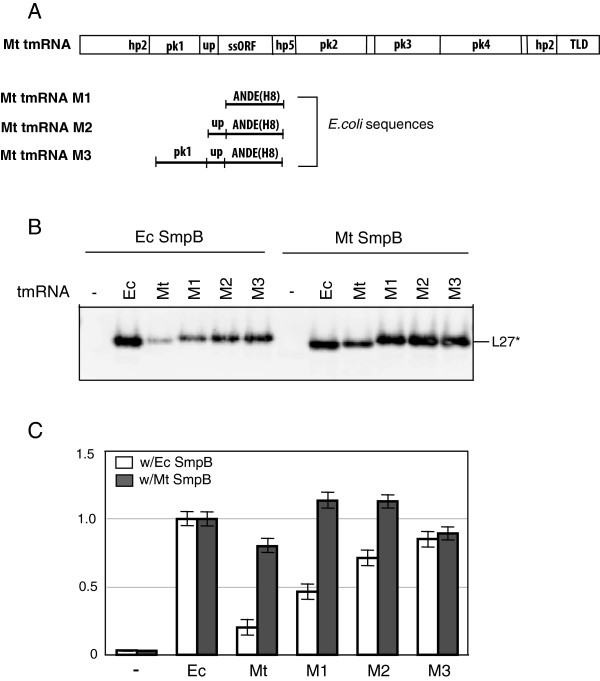
***In vitro *tagging of a truncated ribosomal protein L27 by mutant *M. tuberculosis *tmRNAs in the presence of *E. coli *and *M. tuberculosis *SmpB proteins. (A)** Chimeric tmRNAs M1, M2 and M3 were created by replacing three segments of *M. tuberculosis* tmRNA with corresponding segments of *E. coli* tmRNA. Four pseudoknots, the single-stranded portion of the open reading frame, the tRNA-like domain, helix 2 and helix 5 are denoted pk1-pk4, ssORF, TLD, hp2 and hp5, respectively. The single-stranded sequence connecting pk1 and ORF is marked as ‘up’. ORFs of *E. coli* and *M. tuberculosis* tmRNAs encode ANDE(H8) and ADSHQR(H6) peptide tags, respectively. **(B)***In vitro* tagging of truncated ribosomal protein L27 by chimeric tmRNAs M1, M2 and M3 in the presence of either *E. coli* or *M. tuberculosis* SmpB proteins. Tagging was visualized by Western blotting with anti-T7 tag antibodies in ECL-Plex system. L27* denotes tagged ribosomal protein L27. **(C)** Graphical representation of Typhoon-derived data derived from four Western blotting analyses. Tagging efficiencies of hybrid tmRNA derivatives were normalized and compared to the tagging efficiency of the *E. coli* tmRNA(H8). Error bars show the standard deviation of three or more independent experiments.

**Figure 6:**
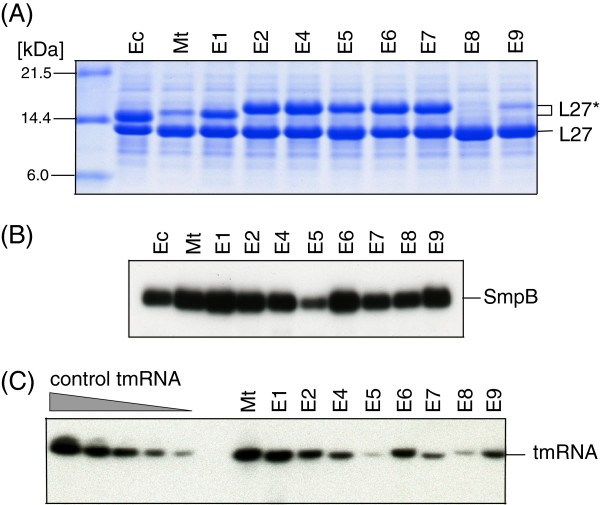
***In vivo *tagging of truncated ribosomal protein L27 by hybrid tmRNAs in the presence of *E. coli *SmpB protein. (A)** Lysates of cells expressing *E. coli* (Ec), *M. tuberculosis* (Mt) and hybrid tmRNAs (E1-E9) were fractionated on a 12.5% SDS polyacrylamide gel. Truncated and tagged proteins were detected by staining the gel with Coomassie Blue. L27 and L27* denote truncated and tagged ribosomal protein L27, respectively. Molecular markers of 6.0, 14.4 and 21.5 kDa are shown on the left side of the gel image. **(B)** Western analysis of the ribosome-bound *E. coli* SmpB. Aliquots of 1.0 A_260_ units of 70S ribosomes derived from lysates of IPTG-induced *E. coli* IW764 cells harboring plasmid pWOW derivatives were fractionated on a 10% SDS-polyacrylamide gel. The SmpB protein was visualized by Western blotting with polyclonal anti-*E. coli* SmpB antibodies. **(C)** Northern analysis of the ribosome-bound tmRNAs present in the tagging reaction mixture. Aliquots of 0.5 μg RNA extracted from 70S ribosomes were separated on a 6% denaturing polyacrylamide gel and blotted to a Zeta-probe membrane. [5’-^32^P]-labeled oligonucleotide probes complementary to a segment of *M. tuberculosis* tmRNA were hybridized to each tmRNA. To estimate quantity of tmRNA in the tmRNA:ribosome complexes, increasing amounts of purified *M. tuberculosis* tmRNA(H6) (0.0125, 0.025, 0.05, 0.1 and 0.2 pmoles) were also fractionated.

**Figure 7:**
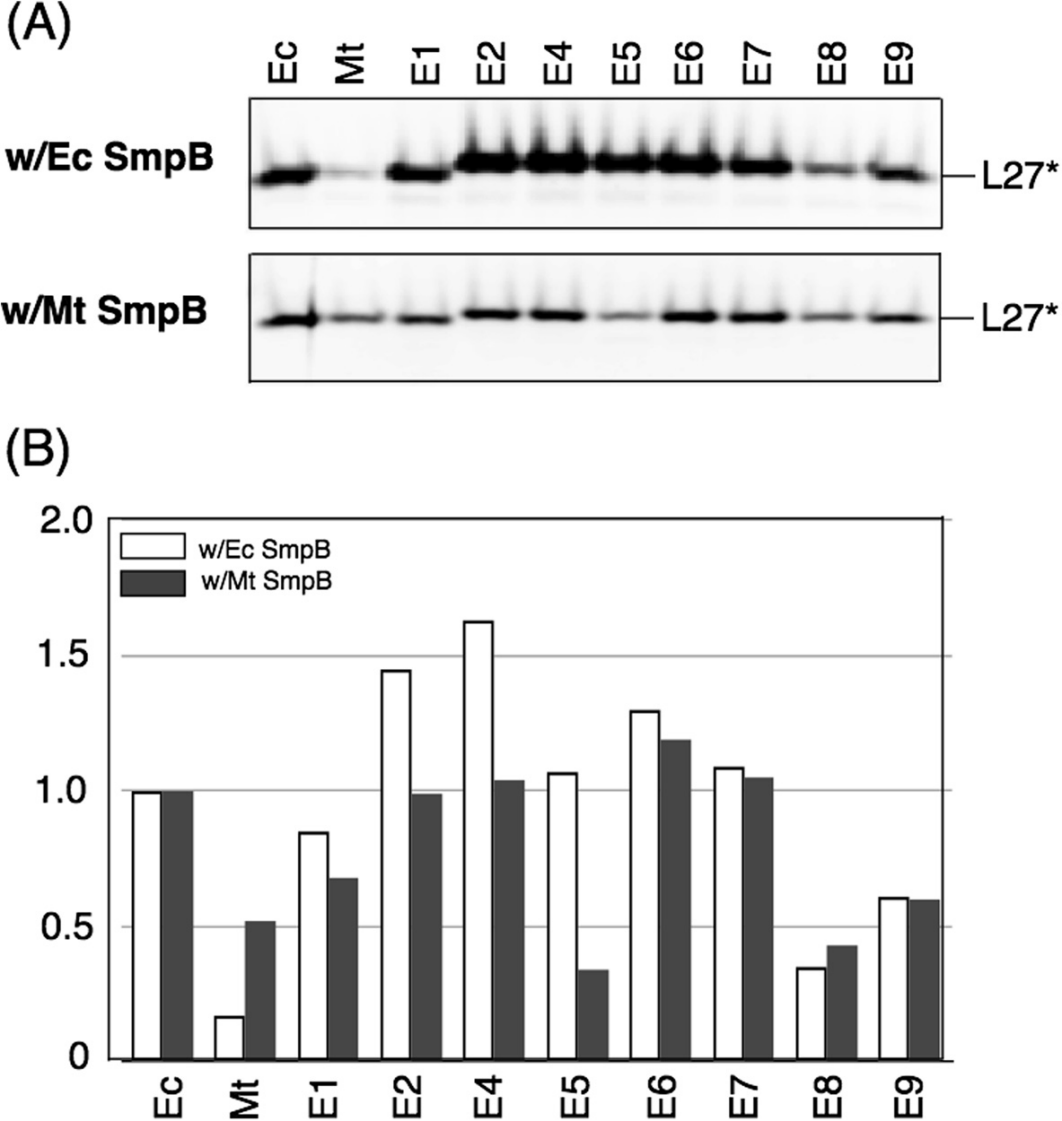
***In vitro *tagging of truncated ribosomal protein L27 by hybrid tmRNAs in the presence of either *E. coli *or *M. tuberculosis *SmpB proteins. (A)** Western Blot analysis. Tagging reactions were assembled by addition of circular plasmid pETrpmA-At encoding truncated protein L27, purified SmpB proteins and tmRNAs to the T7 S30 transcription/translation mixture (Promega). After 60 min incubation at 37 ^o^C, tagged proteins were captured on Ni^2+^-NTA-Sepharose and then fractionated on a 10% SDS-polyacrylamide gel. Tagging was visualized by Western blotting with anti-T7 tag antibodies in ECL-Plex system. L27* denotes tagged ribosomal protein L27. **(B)** Graphical representation of Typhoon-derived data from the Western blot. Tagging efficiencies of hybrid tmRNA derivatives were normalized and compared to the tagging efficiency of the *E. coli* tmRNA(H8):SmpB complex. Error bars show the standard deviation of three or more independent experiments.

To identify regions of the *M. tuberculosis* tmRNA that impair its tagging activity in the presence of *E. coli* SmpB, we constructed 12 chimeric tmRNAs (E1-E9 in Figure 4; M1-M3 in Figure 5; see also Additional file 1: Table S2). All chimeras contained *M. tuberculosis* sequences within the hp5-pk2-pk3 segment known to have no role in the binding to SmpB. *In vivo* tagging activities of chimeric tmRNA:*E. coli* SmpB complexes were analyzed by SDS-PAGE (Figure 6A). As *E. coli* tmRNA(H8) encodes a ten amino acid-long tag ANHHHHHHHH (Figure 1A), its activity produced a fast-migrating L27 protein derivative (Figure 6A, lane 1). *M. tuberculosis* tmRNA(H6) and chimeric tmRNA E1, both encoding a 12-mer ADSHQRHHHHHH (see Figure 1B), produced slower-migrating L27 protein derivatives that readily bind to Ni-NiTA magnetic agarose beads (see also Figure 7A). Unexpectedly, well-tagging chimeric tmRNAs E2-E7, all of which encode a 12-mer ANDEHHHHHHHH, produced abnormally slow-migrating L27 derivatives. The abnormally slow mobility of the ANDEHHHHHHHH-tagged L27 protein in a polyacrylamide gel can be explained by an enhanced tendency of protein segments containing histidyl residues to form a helical structure in the presence of SDS [27] and by an intimate link between increased SDS binding to helical structures [28].

Coomassie Blue stained gels were scanned, the tagging efficiencies of chimeric tmRNAs were measured and compared to the tagging efficiency of *E. coli* tmRNA(H8) as described in Methods (Figure 4). Figure 6A shows that replacing either pk1 or hp2b-2d reduces the *in vivo* tagging activity of E6 and E7 by 12 to 14 percent. Replacing either the single-stranded portion of *E. coli* ORF (ssORF) or the single-stranded sequence upstream of the ORF with the equivalent *M. tuberculosis* segments yielded E3 (data not shown) and E5 that displayed approximately 70 percent tagging activity of *E. coli* tmRNA (H8) (see also Additional file 1: Table S2). The most dramatic decrease of *in vivo* tagging was observed in E8 and E9 that have either *M. tuberculosis* hp2b-2d and pk1 or *M. tuberculosis* pk1 and single-stranded nucleotides upstream the ORF. This finding was unexpected because replacing individually hp2b-2d, pk1 and the single-stranded sequence upstream of the ORF had a relatively small effect on the tagging *in vivo* (Figure 6A, lanes E5-E7).

To determine whether the tagging inefficiencies of *M. tuberculosis* tmRNA(H6) and chimeric tmRNAs E8 and E9 are caused by their poor binding to stalled ribosomes, we measured the ribosomal occupancies of *E. coli* SmpB and tmRNA. Western blotting analysis demonstrated that the inefficient tagging of *M. tuberculosis* tmRNA(H6), and chimeric tmRNAs E8 and E9 was not caused by the inability of *E. coli* SmpB to associate with the ribosome (Figure 6B). Similar amounts of the ribosome-bound SmpB were found in the efficiently tagging *E. coli* tmRNA (H8) and the extremely poorly tagging chimeric tmRNA E8 preparations. Northern blot analysis of the ribosome-bound tmRNAs produced a difficult to interpret yet reproducible profile (Figure 6C). Ribosomal occupancies of the poorly tagging *M. tuberculosis* tmRNA (H6) and the efficiently tagging chimeric tmRNA E1 were similarly pronounced while ribosomal occupancies of the poorly tagging chimeric tmRNA E8 and the well tagging E5 were similarly reduced. This may be explained by a scenario where the tmRNA E5 is either readily degraded or quickly cleared off the *trans*-translating ribosomes.

### *In vitro* tagging activity of chimeric tmRNAs

In order to characterize the chimeric tmRNAs further, we analyzed their tagging properties *in vitro*. These assays were carried out in the *E. coli* T7 transcription/translation system for circular DNA (Promega) and employed the plasmid pET*rpmA*-At-1 that encodes truncated L27 [24]. Reaction mixtures were supplemented with purified transcripts of chimeric tmRNAs and purified SmpBs from either *E. coli* or *M. tuberculosis*.

The *in vitro* tagging pattern of chimeric tmRNA:*E. coli* SmpB complexes is shown in Figure 7A. As demonstrated *in vivo*, E8 and E9 tag truncated ribosomal protein L27 poorly (<1 to 9 percent) at levels slightly higher than the degree of tagging observed with *M. tuberculosis* tmRNA(H6). This observation confirms that as yet uncharacterized structural features in the segment of *M. tuberculosis* tmRNA encompassing hp2b-2d, pk1 and the single-stranded sequence upstream of the ORF are responsible for the tagging inefficiency of *M. tuberculosis* tmRNA:*E. coli* SmpB complexes.

Figure 7A shows that the chimeric tmRNAs tag at different levels when analyzed *in vitro* in the presence of *M. tuberculosis* SmpB. Quantitative analysis of data derived from the *in vitro* experiments shows that *M. tuberculosis* SmpB is more effective in supporting the tagging activity of *E. coli* tmRNA(H8) than *M. tuberculosis* tmRNA(H6) (Figure 7B). Given that E5 is the least active chimeric tmRNA, the inefficient tagging of homologous *M. tuberculosis* tmRNA(H6):SmpB complexes on *E. coli* stalled ribosomes is likely caused by the single-stranded nucleotides upstream of the ORF. This observation is in conflict with an earlier study showing that replacing 85-UAGUC-89 with 85-UAAGC-89 in *E. coli* tmRNA does not affect the activity of the mutant tmRNA in the KanR assay [20].

### Tagging activity of *M. tuberculosis* tmRNAs derivatized with segments of *E. coli* tmRNA

As demonstrated above, replacing hp2b-2d, pk1, the single-stranded sequence upstream of the ORF and the ssORF in *E. coli* tmRNA with equivalent segments of *M. tuberculosis* tmRNA affects, to a variable extent, tagging activities of chimeric tmRNAs E3, E5, E6 and E7 (Figure 4). Results obtained using chimeric tmRNAs M1, M2 and M3, constructed by replacing pk1, the single-stranded sequence upstream of the ORF and the ssORF in *M. tuberculosis* tmRNA with equivalent segments of *E. coli* tmRNA, were equally revealing (Figure 5A). As seen in Figures 5B and 5C, replacing the ssORF in *M. tuberculosis* tmRNA with the equivalent segment of *E. coli* tmRNA had the most dramatic effect on tagging activities of the resulting chimeric tmRNAs M1, M2 and M3. When tested with *E. coli* SmpB, M1, M2 and M3 displayed respectively 2.4, 3.9 and 4.6-fold higher tagging activities than *M. tuberculosis* tmRNA(H6). In contrast, the chimeric tmRNA M1 was the most active when tagged *in vitro* in the presence of *M. tuberculosis* SmpB (Figures 5B and 5C). The gradual increase of the tagging activity in chimeric tmRNAs M1 and M2, and the inefficient tagging displayed by the chimeric tmRNA E8 derivative in the presence of *E. coli* SmpB suggest that the structural relationship between pk1 and the sequence upstream of the ORF is an important determinant for establishing proper interactions within the *E. coli* tmRNA:SmpB complex. Because the tag template after the +2 codon can be replaced by many different sequences without any loss of activity [24,29], it seems that the efficient tagging activity of the chimeric tmRNA M1: *M. tuberculosis* SmpB complex on *E. coli* ribosomes results from replacing the GCA-AAC sequence of the resume and +2 codon with the GCC-GAU sequence. Because changing the GCA triplet for the GCC triplet in the resume codon has very little effect on *E. coli* tmRNA tagging activity, the first nucleotide of the +2 codon is likely to enhance the tagging activity of chimeric tmRNA E1 *in vitro*[30]. This is consistent with a conservation of adenosine residues in the first two positions of the +2 codon [31].

## Discussion

The *ssrA* gene is present in all known bacterial and some organellar genomes [32,33]. Its transcription produces single-chain tmRNA molecules in most bacteria and two-piece tmRNAs in certain major lineages that share similar architectures [34,35]. The most conserved features include the tRNA-like domain (TLD) and mRNA-like region (MLR) with an open reading frame (ORF). As shown in Figure 1, they are connected to each other by a number of short helices (hp2a-2d) and four pseudoknots (pk1-pk4). The TLD, hp2d, part of pk1, a number of nucleotides in the single-stranded sequence upstream of the ORF and the resume codon are highly conserved [36]. In contrast, the nucleotide composition and the length of the ORF can be highly variable. Because only the resume and +2 codons display biases for certain nucleotide triplets, a large portion of the ORF could be replaced by unrelated sequences without affecting the tagging activity of tmRNA [24,31]. Such conservation pattern suggests that tmRNAs, like canonical tRNAs, might be able to support protein synthesis in the distantly related bacteria. Results derived from our experiments with tmRNAs derived from *E. coli* (Proteobacteria) and *M. tuberculosis* (Actinobacteria) support this idea. Both tmRNA molecules tag, albeit with different efficiencies, truncated proteins in *E. coli. M. tuberculosis* SmpB effectively promotes tagging activities of both the *E. coli* and the *M. tuberculosis* tmRNAs. In contrast, *E. coli* SmpB supports the tagging activity of *M. tuberculosis* tmRNA very poorly. Our present studies demonstrate that replacing the hp5-pk2-pk3-pk4 segment of *E. coli* tmRNA with the equivalent segment from *M. tuberculosis* tmRNA have very little effect on the tagging activity of the resulting chimeric tmRNA E2 molecule. This is consistent with earlier studies, which demonstrated that extensive changes could be introduced into hp5 without significant effect on *E. coli* tmRNA tagging activity as long as the base pairing of hp5 is maintained [24]. While interchanging pk2, pk3 and pk4 causes only minor losses in tagging activity of mutant tmRNAs, disrupting the structure of these three pseudoknots has a differential effect not only on tmRNA tagging activity but also on its maturation [24,25]. Together, these findings indicate that the role of pk2, pk3 and pk4 is to maintain proper overall folding of the tmRNA molecule.

Replacing the ssORF in *E. coli* tmRNA with the equivalent segment of *M. tuberculosis* tmRNA reduces its tagging activity by about 30 percent. In contrast, replacing the ssORF in *M. tuberculosis* tmRNA with the equivalent segment of *E. coli* tmRNA increases its tagging activity to about 130 percent of the activity observed with an unmutated *M. tuberculosis* tmRNA. The sequence comparison of the ORF region in all known tmRNAs shows conservation only in the resume codon and the +2 codon [31]. Therefore, the increased tagging activity of the chimeric tmRNA M1, which uses GCA instead of GCC as its resume codon, can be explained by the differences in the availability of tRNA isoacceptors that decode these two codons. According to earlier studies, the GCA triplet is decoded by Ala-tRNA_1B_ that belongs to the most abundant tRNAs in *E. coli* and constitute about five percent of the total tRNA. In contrast, the GCC triplet is decoded by the tRNA_2_ isoacceptor that constitutes only about one percent of the total tRNA [37,38].

The importance of the +2 codon for tmRNA functions is highlighted by studies of O’Connor [39] and Thibonnier et al. [40]. They demonstrated that translation of the resume codon alone is sufficient for both adding a minimal tag of two amino acids to a truncated protein and tmRNA-dependent ribosome recycling in *E. coli,* respectively. However, when two stop codons are introduced immediately downstream from the resume codon, the resulting mutant tmRNA is unable to complement the slow growth phenotype of *E. coli* lacking the chromosomal copy of the *ssrA* gene. When tagging activity of *M. tuberculosis* tmRNA is tested in *E. coli*, it is possible that the +2 codon (GAU) compensates a poor resume codon (GCC). This compensation relates most likely to the GAU-decoding tRNA_1_^Asp^ that is more abundant (3.72%) than the AAC-decoding tRNA^Asn^ (1.85%) [38]. However, additional studies are required to assess the contribution of the +2 codon to the tagging activity of the tmRNA molecule in general and to explain why adenosine is preferred at the second position of the +2 codon in particular [31].

The properties of pk1 and the single-stranded sequence immediately upstream the ORF have been studied extensively. Although residues 49–53 and 64–72 (*E. coli* tmRNA numbering; see Figure 1A) of pk1 are conserved [36], replacing pk1 with a single-stranded RNA yields a mutant tmRNA derivative that tags truncated proteins efficiently *in vivo*[22]. TmRNA sequence comparisons showed a strong preference for AUAG and AUAA tetramers upstream of the resume codon [41]. Mutations in these tetramers lead to either -1 or +1 frameshifting [23,42,43]. Given that both *E. coli* and *M. tuberculosis* tmRNAs exhibit the same sequence preference, a 27% drop in tagging activity of the chimeric tmRNA E5, which has *M. tuberculosis* single-stranded upstream sequence, was unexpected. Equally unexpected was either total or almost total tagging inactivity of *E. coli* tmRNA mutants having *M. tuberculosis* segments composed either of helices 2b-2d and pk1 or of pk1 and the single-stranded segment upstream of the ORF. Because sequences forming hp2b and hp2c are poorly conserved and because both *E. coli* and *M. tuberculosis* pk1 and single-stranded upstream sequence share almost all of the conserved nucleotide residues, the observed tagging defect must be induced by changes in the structure of the linker formed by hp2, pk1 and a single-stranded upstream sequence. Such changes are likely to affect the binding of SmpB protein to tmRNA. Two copies of this protein are able to bind to a single tmRNA molecule both on and off the ribosome [16,44,45]. One of them interacts with and cross-links to the T loop of the tmRNA [44]. This interaction is believed to play a minor, if any, role in tmRNA functions on stalled ribosomes. In contrast, the second SmpB molecule interacts with two segments of the ribosome-bound tmRNA. X-ray analysis revealed that it binds to the TLD to play a role of the missing D arm and its C-terminal part of beta 7 strand mimics the anticodon loop [6]. Moreover, the conserved residues of the beta 5 strand of the second SmpB molecule orient hp2A toward the decoding site of the 30S ribosomal subunit. According to cryo-EM studies, when the TLD is accommodated in the P site, the 85-UAGUC-99 pentamer in the single-stranded upstream sequence is in proximity to and most likely interacts with the residues 134–140 in the C-terminus of *E. coli* SmpB [18]. As established by a number of earlier studies, this interaction is essential for a proper selection of the resume codon [41,42,46]. Our present work revealed that changes in the linker region are able to inactivate the tagging activity of tmRNA. Given that both *M. tuberculosis* and *E. coli* SmpB proteins support the tagging activity of *M. tuberculosis* tmRNA, one can speculate that the structure of its hp2-pk1-upstream sequence linker better accommodates the requirements of both SmpB proteins. Future studies will reveal in detail the structural features in the linker region that tmRNA needs to support efficient *trans*-translation.

## Conclusion

This study significantly advances our understanding of *trans*-translation by demonstrating the existence of yet uncharacterized structural constraints in helices 2b-2d, pk1 and the single-stranded sequence upstream of the ORF. Although these conserved segments of tmRNA are not believed to interact directly with SmpB, they have a profound effect on the tagging activity of tmRNA:SmpB complexes. Moreover, our finding that tmRNA and SmpB can function in heterologous systems extends the investigation of *trans*-translation beyond the standard *E. coli* model and provides opportunities to target the *trans*-translaton system of *M. tuberculosis* for pharmacological intervention against multidrug-resistant tuberculosis [11,47].

## Methods

### Bacterial strains and plasmids

*E. coli* strain XL1-B was the host for cloning. Expression strain IW764 was derived from *E. coli* BL21(DE3)/pLysS by deleting *smpB* and *ssrA* genes. DNA fragment from *Bst*XI restriction site in *smpB* and *Sph*I in *ssrA* was replaced with the kanamycin-resistance gene from plasmid pACYC177. IW764 strain was constructed as described in [48].

Plasmids used in the study are listed in Table S1. Master plasmid pWOW was produced by modifying the pETrpmA-At-3 plasmid [24]. It has additional restriction sites at the start (*Nde*I) and at the end (*EcoR*I) of the *smpB* gene. These sites were used to replace *E. coli smpB* gene with *M. tuberculosis smpB* gene*.* Plasmid pWOW variants containing *M. tuberculosis ssrA* gene were constructed using 2-step PCR protocol described earlier [49]. DNA fragment encoding mature *E. coli* tmRNA was replaced with equivalent mature *M. tuberculosis ssrA* gene. *M. tuberculosis smpB* and *ssrA* genes remained under control of *E.coli* regulatory signals. *E. coli* and *M. tuberculosis smpB* genes were cloned between *Nde*I and *Xho*I restriction sites in protein expression vector pET-23a. Overexpressed SmpB proteins have an additional LEHHHHHH tag at their C-termini.

### Purification of SmpB proteins

*E. coli* and *M. tuberculosis* SmpB proteins were overexpressed in the IW764 strain. *E. coli* cells were incubated at 30°C and protein expression was induced in the mid-log phase following standard protocols. Proteins were extracted with the lysis buffer containing 100 mM sodium phosphate, 10 mM Tris, 8 M urea (pH 8.0) and purified on Ni^2+^-NTA-agarose (Qiagen) under denaturing conditions and dialyzed against the storage buffer containing 50 mM MES-KOH (pH 6.5), 200 mM KCl, 5 mM 2-mercaptoethanol and 10% glycerol. This procedure yields His-tagged SmpB proteins that are >95% pure.

### Synthesis and purification of chimeric tmRNAs

Plasmid ptmR with the *ssrA* gene derivatives under control of the T7 promoter was linearized with restriction enzyme *Bst*NI and used as a template for the *in vitro* synthesis of chimeric tmRNAs as described earlier [10]. TmRNA transcripts were purified on RNeasy mini-spin columns according to instructions provided by the manufacturer (Qiagen). The integrity of the transcripts was tested by electrophoresis on a denaturing 5% polyacrylamide gel.

### *In vivo* tagging assay

To monitor *in vivo* tagging of chimeric tmRNAs, we modified an earlier described procedure [24]. *E. coli* IW764 cells were transformed with plasmid pWOW coding for truncated protein L27 under control of T7 promoter and a combination of *E. coli* or *M. tuberculosis* SmpB and tmRNA variants under the control of native *E.coli* promoters. Cells were grown at 37°C in 2xYT broth supplemented with ampicillin (200 mg/mL), chloramphenicol (30 mg/mL) and kanamycin (50 mg/mL). 1 mM IPTG was added when A_600_ reached 0.3. After additional 3 hours of incubation, cells were collected by low-speed centrifugation. Cell pellets for RNA analysis were flash-frozen and stored at -80°C. Cells for protein analysis were lysed in a SDS-polyacrylamide gel-loading buffer at a concentration of 0.005 A_600_/μL. 5–10 μL aliquots of lysates were fractionated on a 12.5% SDS/Tricine-polyacrylamide gel and stained with Coomassie Brilliant Blue R-250 (Sigma).

### *In vitro* tagging assay

All assays were performed with the *E. coli* T7 transcription/translation system for circular DNA from Promega [22]. A typical 25-μL reaction mixture contained 2 μg of circular plasmid pETrpmA-At-1 encoding the gene for truncated ribosomal protein L27, 25 pmoles of His-tagged SmpB protein, 40 pmoles of tmRNA, and 20 units of SUPERase-In^TM^. Tagging reaction was carried out for 1 h at 37°C. Tagged proteins were captured on Ni-NTA magnetic agarose beads (Qiagen), fractionated on a 10% SDS/Tricine-polyacrylamide gel and blotted to a Hybond-LFP membrane (GE Healthcare Life Sciences). Because His-tagged truncated protein L27 had T7-tag peptide on its N-terminus, it could be detected using the anti-T7 tag monoclonal antibody from Invitrogen and the secondary Cy5-labeled antibody from Amersham. Band intensity was quantified using a Typhoon Phosphoimager and ImageQuant software (Amersham Biosciences).

### Detection of tmRNA in cell lysates

Total bacterial RNA was extracted using RNeasy kit (Qiagen). Its concentration was measured using Quant-iT RNA assay kit from Invitrogen. Aliquots of 0.5 to 1 μg of total RNA were fractionated on a denaturing 5% polyacrylamide gel. RNA was blotted to a Zeta-probe membrane (Bio-Rad). Blots were probed with [5’- ^32^P]-labeled oligonucleotides 5’-CAGCTGCGGACGGACAC-3’ and 5’-GTGAGTCCCTCTAGCTG-3’ complementary to *E. coli* tmRNA(H8) and *M. tuberculosis* tmRNA(H6), respectively. Hybridization signals were visualized using a Typhoon 9410 Phosphoimager and ImageQuant software (GE Healthcare).

TmRNA:70S ribosome complexes were purified as described previously [19].

### Detection of protein SmpB in cell lysates

Lysates of 0.02-0.03 A_600_ units of cells were fractionated on a 10% SDS/Tricine-polyacrylamide gel. Proteins were blotted to a Hybond-LFP membrane (GE Healthcare) by a wet transfer in Towbin buffer containing 20% methanol and 0.05% SDS. Rabbit polyclonal antibodies raised against the *E.coli* SmpB protein and Cy5-labeled anti-rabbit secondary antibodies from ECL-Plex system (Amersham, GE Healthcare) were used to detect both *E.coli* and *M. tuberculosis* SmpB proteins. Blots were visualized using Typhoon 9410 or LAS 4100 imagers and quantified using ImageQuant software (GE Healthcare).

SmpB proteins with the Tag-100 at their C-termini were detected with rabbit anti-Tag-100 monoclonal antibody from GenScript and anti-rabbit IgG-HRP using the ECL-Plus Western Blotting System (Amersham, GE Healthcare).

## Competing interests

The authors declare that they have no competing interests.

## Authors’ contributions

IKW developed assays for testing the tagging activities of chimeric tmRNAs in the presence of either *E. coli* or *M. tuberculosis* SmpB proteins. CZ conceived the study and participated in all of its aspects. JW cloned, overexpressed and purified *E. coli* and *M. tuberculosis* SmpB proteins. All authors wrote and approved the manuscript.

## Supplementary Material

Additional file 1: Table S1 Bacterial strains and plasmids. **Table S2.** Engineering chimeric tmRNAs. **Figure S1.** pWOW plasmid. Click here for file
