# Supplementary material for: Requirements for resuming translation in chimeric transfer-messenger RNAs of Escherichia coli and Mycobacterium tuberculosis
Source: BMC Mol Biol. 2014 Sep 15;15:19. doi: 10.1186/1471-2199-15-19 (PMC4236655; doi:10.1186/1471-2199-15-19)
Supplement: Additional file 1: Table S1 — Bacterial strains and plasmids. Table S2. Engineering chimeric tmRNAs. Figure S1. pWOW plasmid. [file 1471-2199-15-19-S1.pdf]

Table S1. Bacterial strains and plasmids .

Strain/plasmid	Properties	Reference
<u>*Escherichia coli* strains</u>		
XL1-B	wild type	
BL21(DE3)/pLysS	wild type	
IW764	BL21(DE3)/pLysS, Δ(*smpB-ssrA*)::*kan*^{r})	this paper
<u>*Mycobacterium tuberculosis*</u>		
serovar H37Rv	source of *ssrA* and *smpB* genes	
<u>Plasmids</u>		
pET*rpmA*-At-3	master plasmid for tagging *in vivo*	(25)
	rpmA-At-t, *smp*B(*Ec*), *ssr*A(*Ec*), *amp*^{r},	
pWOW-E/E	derivative of pETrpmA-At-3 with *Nde*I	
	restriction site at 5'-end of *smp*B gene	this paper
pWOW-Δ/E	Δ*smpB, ssrA*(*Ec*)	this paper
pWOW-M/E	*smpB*(*Mt*), *ssrA*(*Ec*)	this paper
pWOW-Δ/M	Δ*smpB, ssrA*(*Mt*)	this paper
pWOW-M/M	*smpB*(*Mt*), *ssrA*(*Mt*)	this paper
pWOW-E/M	*smpB*(*Ec*), *ssrA*(*Mt*)	this paper
ptmR	*ssrA* gene from *E.coli* or variants	(10)
	under T7 promoter control	
pET-*smpB*	expression vector, *smpB*(*Ec* or *Mt*)	this paper

Table S2: Engineering chimeric tmRNAs.

Chimeric Ec tmRNA	Ec tmRNA(H8) nucleotides removed	Mt tmRNA(H6) nucleotides inserted
E1	90 - 299	95 - 304
E2	108 - 299	116 - 304
E3	90 - 245	95 - 246
E4	108 - 245	116 - 246
E5	79 - 89	83 - 94
	108 - 245	116 - 246
E6	49 - 78	48 - 82
	108 - 245	116 - 246
E7	34 - 48	33 - 47
	108 - 245	116 - 246
	303 - 320	308 - 324
E8	49 - 89	42 - 94
	108 - 245	116 - 246
E9	34 - 78	33 - 82
	108 - 245	116 - 246

Chimeric Mt tmRNA	Mt tmRNA(H6) nucleotides removed	Ec tmRNA(H8) nucleotides inserted
M1	95 - 115	90 - 107
M2	83 - 115	79 - 107
M3	48 - 115	49 - 107

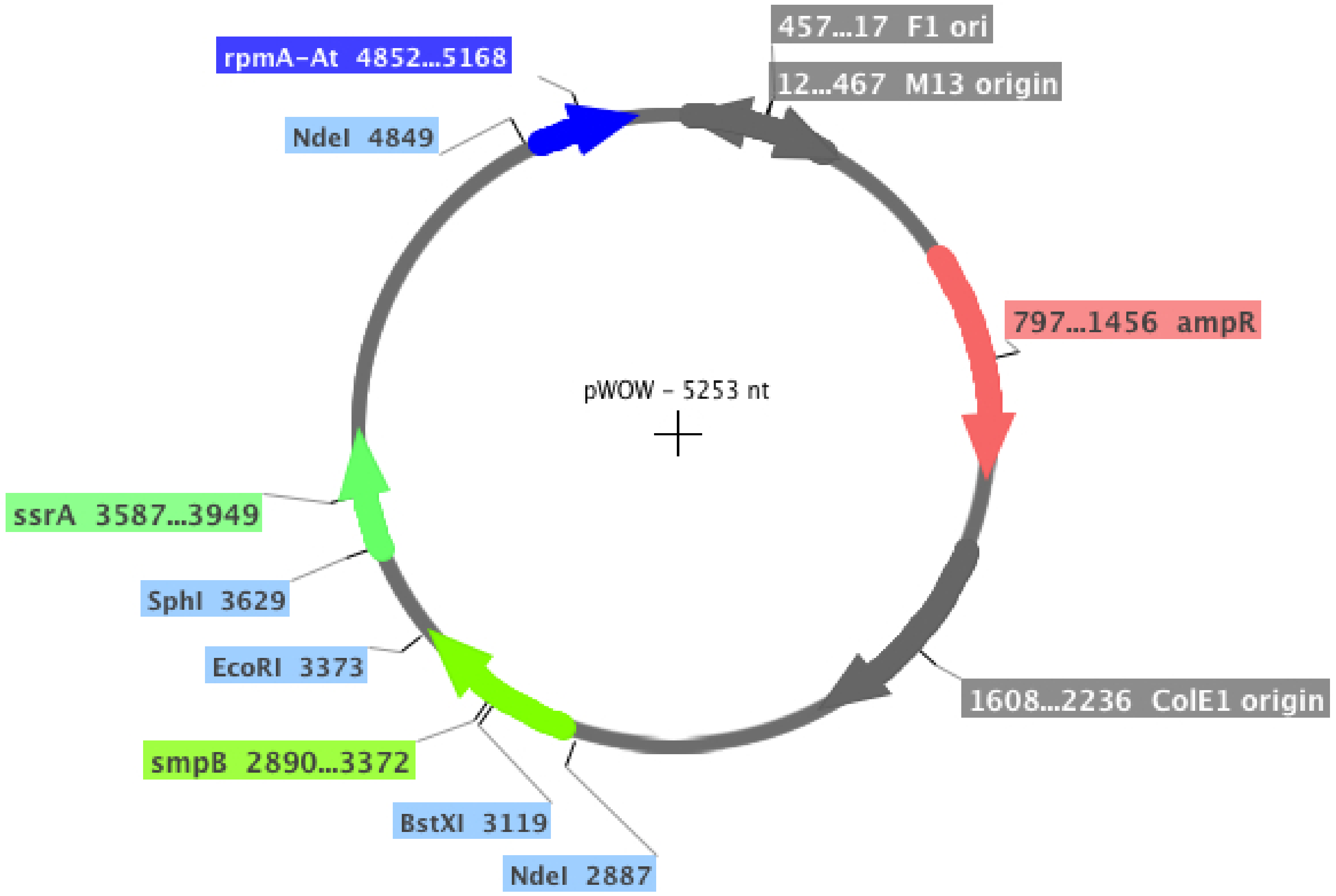

457...17 F1 ori
12...467 M13 origin
rpmA-At 4852...5168
NdeI 4849
797...1456 ampR
pWOW - 5253 nt
ssrA 3587...3949
SphI 3629
EcoRI 3373
1608...2236 ColE1 origin
smpB 2890...3372
BstXI 3119
NdeI 2887

Fig. S1
